# Change in *Anopheles* richness and composition in response to artificial flooding during the creation of the Jirau hydroelectric dam in Porto Velho, Brazil

**DOI:** 10.1186/s12936-017-1738-7

**Published:** 2017-02-22

**Authors:** Moreno S. Rodrigures, Elis P. Batista, Alexandre A. Silva, Fábio M. Costa, Verissimo A. S. Neto, Luiz Herman S. Gil

**Affiliations:** 1 Universidade Federal de Rondônia, BR 364, Km 9.5, CEP 76800-000 Porto Velho, RO Brazil; 2 Universidade Federal de Mina Gerais, AV. Antônio Carlos, 6627, CEP 31270-901 Belo Horizonte, MG Brazil; 3 Energia Sustentável do Brasil, Av. Joaquim Nabuco, 3200 Areal, CEP 76801-066 Porto Velho, RO Brazil; 4 Instituto de Patologia em Doenças Tropicais, Rua da Beira 7671, CEP 76812-245 Porto Velho, RO Brazil

**Keywords:** Amazon, Malaria, Human activity, Vector ecology

## Abstract

**Background:**

*Anopheles* mosquitoes are the only vectors of human malaria. *Anopheles* species use standing water as breeding sites. Human activities, like the creation of an artificial lake during the implementation of hydroelectric power plants, lead to changes in environmental characteristics and, therefore, may changes the species richness and composition of *Anopheles* mosquitoes. The aim of the present study was to verify whether or not there is an association between the artificial flooding resulting from the construction of the Jirau hydroelectric power plant, and the richness and composition of anophelines.

**Methods:**

Mosquitoes samples were obtained monthly from the Jirau hydroelectric power plant area located at Porto Velho, Rondônia State, using Human Landing Catch (06:00–10:00 PM). Mosquitoes collected were transported to Laboratório de Entomologia Médica FIOCRUZ-RO where they were identified until species using dichotomous key.

**Results:**

A total of 6347 anophelines belonging to eight different species were collected. The anophelines species richness was significantly lower during the first flooding stage. Differences in anophelines species composition were found when comparing the first flooding stage with the other stages. Furthermore, the mean number of *Anopheles darlingi*, the main vector of malaria in the region, increases during the first and the third flooding stages.

**Conclusions:**

The continual monitoring of these vectors during the late operational phase may be useful in order to understand how anophelines will behave in this area.

**Electronic supplementary material:**

The online version of this article (doi:10.1186/s12936-017-1738-7) contains supplementary material, which is available to authorized users.

## Background

Blood-sucking mosquitoes (Diptera: Culicidae) are the main vectors of human diseases, including dengue, yellow fever, zika, chickungunya and malaria. Even non-vector mosquitoes can be extremely aggressive when blood-feeding which can cause a great nuisance to their hosts. Thus, this group has an important impact on public health [1, 2]. In this scenario, mosquitoes belongings to the Anophelinae subfamily are particularly important. This subfamily is composed of three genera, *Chagasia, Bironella* and *Anopheles*, of which *Anopheles* are the only vectors of *Plasmodium* spp. (the aetiological agents of malaria) to humans.

*Anopheles* mosquitoes are found in tropical and neotropical regions, and up until 2016, 472 species had been recognized [3]. The occurrence of an anopheline species in an endemic area is associated with the environmental characteristics of the region. Thus, important aspects of an anophelines population, such as species richness and composition, are ruled by environmental characteristics, including food availability, breeding sites and vegetation [4].

It is well documented that human activities may cause severe alterations to environmental conditions [5]. This phenomenon occurs throughout the entire world, and, South America is currently one of the regions where human activities have increased drastically. In the Amazon region, activities like family farming, selective logging, and construction of hydroelectric power plants are common [6, 7]. The changes can be different according to the type of activity, but in general they could be classified as primary changes (i.e. alterations in vegetation, water bodies or river’s flow) and secondary changes (i.e. influx or efflux population, increasing in the quantity of potential hosts/reservoir for both vector and pathogen) [8, 9].

In response to the alterations, anopheline species richness and composition can drastically change, thereby altering the malaria dynamic in the region [6]. Thus, the aim of these study was to monitoring the anophelines fauna in the area of the Jirau hydroelectric power plants (UHE-Jirau). Specifically, the study focuses on three main questions regarding the artificial flooding: (i) Is there an increase or decrease in the number of anopheline species? (ii) Is there some alteration in the species composition? and, (iii) Is there an increase or decrease in *Anopheles darlingi* population, the main malaria vector in the region?

## Methods

### Mosquito sampling

From April 2011 to December 2015, anopheline mosquitoes were monthly sampled from seven different sites located in UHE-Jirau, city of Porto Velho in Brazil’s northern region (64°38′31″W, 9°15′52″S) (Additional file 1). The UHE-Jirau is constructed in the Rio Madeira and its’ dam has a total area of approximately 6.5 km, the vegetation in the region is classified as open tropical forest, the mean annual temperature varies from 23 to 25 °C, and the mean regional precipitation is approximately 2500 mm, with 180 rainy days per year. The relative humidity varies from 90% in January to 75% in July, with an annual mean of 85%.

The captures were performed by Human Landing Catch (HLC). As the blood feeding behavior of *Anopheles* has a peak of activity between 07:00 and 09:00 PM [4, 10] during the first 4 hours of the scotophase (06:00–10:00). Sampled mosquitoes were stored in 10 cm high plastic cups, identified with month, day, hour and, location. Then, the mosquitoes were transported to the Laboratório de Entomologia Médica (Medical Entomology Laboratory) of the Instituto de Pesquisas em Patologias Tropicais (Tropical Disease Research Institute) and Oswaldo Cruz Foundation—RO where they were identified to species using a dichotomous key for females.

### Variable description and data analysis

In this study, the water level data of the Madeira River in UHE-Jirau’s area, provided by the Energia Sustentável do Brasil (ESBR) (Sustainable Energy of Brazil), was used as a explanatory variable (x) and in the next section of the text it will be referred as the stage of flooding. The stage of flooding is a categorical variable with four different levels: (i) 75 m, (ii) 84 m, (iii) 90 m corresponding to the first, second and third flooding phases respectively and, (iv) the pre-flood stage, which corresponds to the period before the flooding (artificial lake creation). As response variable (y), *Anopheles* species richness, composition, and *An. darlingi* abundance was used. Species richness was considered as the number of different species sampled at each point and species composition as the proportion of different anophelines species compared to the total at a given point. To compute *An. darlingi* abundance, a simply sum of all the mosquitoes of this specie sampled from a given point was performed.

In order to compare the species richness among stages of flooding, Coverage-based rarefaction and Extrapolation method using Hill’s numbers was used. The Hill’s numbers can be described by the following equation:

$$^qD = \left( {\mathop \sum \limits_{i = 1}^{S} p_{i}^{2} } \right)^{{\frac{1}{1 - q}}}$$

This equation has a very useful property, as q change a different ecological index can be computed so, as q = 0 (species richness), q = 1 (Shannon diversity) and q = 2 (Simpson index).

To evaluate whether or not there is a difference in the species composition among stages of flooding, a Non-Parametric Multivariate Analysis of Variance (PERMANOVA) was use. PERMANOVA was applied on a Bray-Curtis distance matrix calculated on double square root transformed data. Upon detection of a difference with the PERMANOVA model, we then applied a posteriori test corrected for multiple comparison. In order to assess changes in *An. darlingi* abundance among the stages of flooding, a Bayesian generalized mixed model was used. In this model, the stage of flooding was used as a fixed effect and the point was set as a random term. In order to estimate the parameter (i.e. abundance), a Markov chain Monte Carlo (MCMC) with two chains of 20,000 cycles was used. The first 5000 cycles was burned (i.e. discarded) and after this, samples of the posterior distribution were collected from each 5 cycles. To check whether or not the chain converged, trace plot and *Rhat* was used. All analyses were performed using R 3.2.3 [11] with the packages vegan, R2OpenBUGS, iNEXT, coda and, ggplot2. All the data used for us are available as an additional file (Additional file 2).

### Ethical consideration

All participants that performed HLC reviewed and signed a consent term (Free and Clarified Consent Term); the project was approved by the Ethics research committee of the Centro de Pesquisas em Medicina Tropical (CEPEM) (CAAE 0228512.3.0000.0011).

## Results

From April 2011 to December 2015, using the HLC, 6347 anophelinae belonging to 8 different species were collected. Of the mosquitoes sampled, only 267 (4.20%) could not be identified at species level; of the remaining mosquitoes, 5574 were identified as *An. darlingi* (87.80%), 199 as *Anopheles braziliensi*s (3.13%), 124 as *Anopheles triannulatus* (2.00%), 94 as *Anopheles nuneztovari* (1.50%), 47 as *Anopheles konderi* (0.75%), 21 as *Anopheles deaneorum* (0.39%) and, 16 as *Anopehles mattogrossensis* (0.25%) (Table 1).

**Table 1:** *Anopheles* (Diptera: Culicidae) species and abundance among the different flooding stages of UHE Jirau, Porto Velho, Rondonia, Brazil from 2011 to 2015

	Flooding stage				
	Pre	First	Second	Third	Total
*Anopheles braziliensis*	94	8	48	49	199
*Anopheles darlingi*	2223	194	489	2668	5574
*Anopheles deaneorum*	4	0	6	11	21
*Anopheles konderi*	11	0	9	27	47
*Anopheles matogrossensis*	3	0	7	6	16
*Anopheles nuneztovari*	60	10	22	5	97
*Anopheles triannulatus*	44	0	30	44	126
Not identified	0	8	11	256	267

**Fig. 1:**
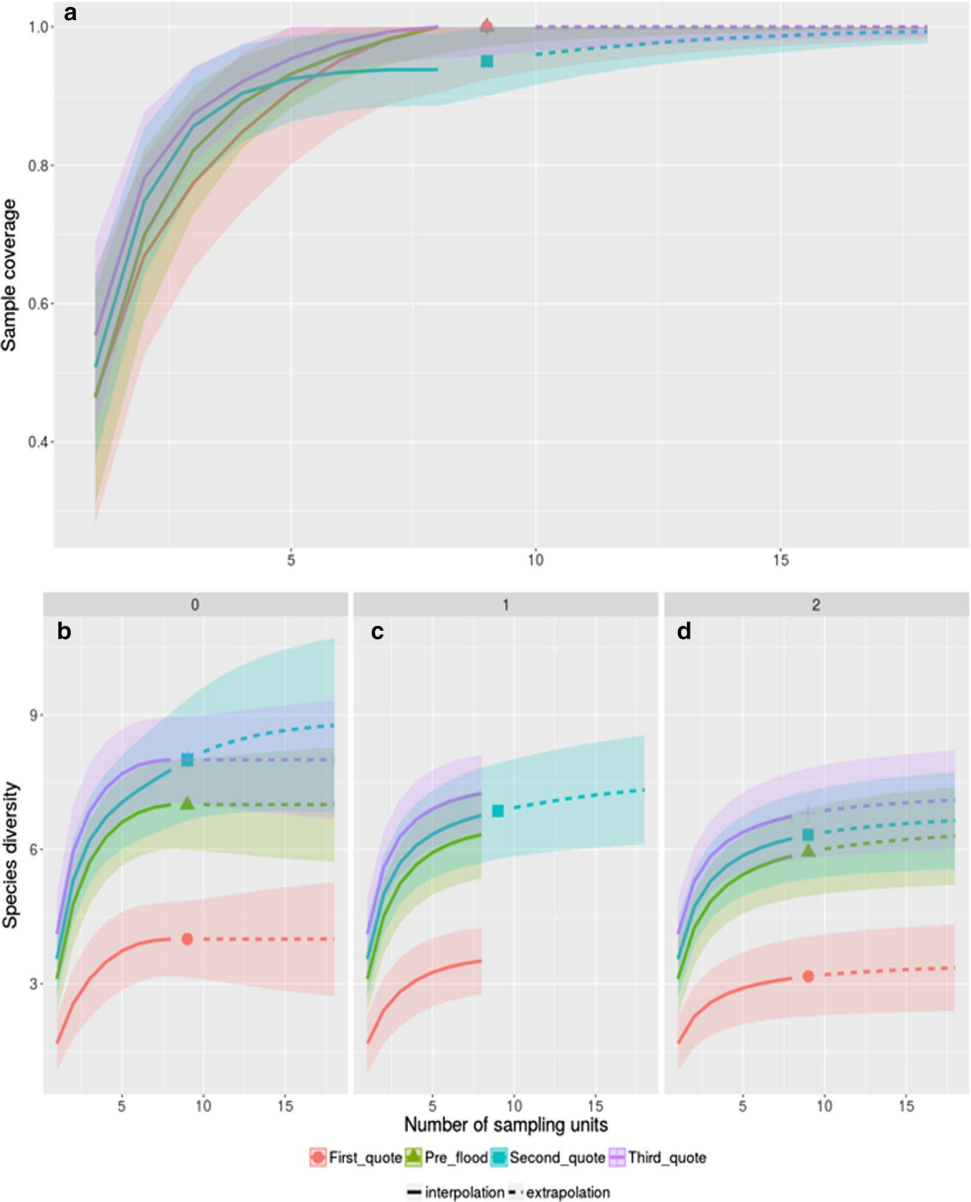
**a** Coverage-based rarefaction (*solid line*) and extrapolation (*dashed line*) plots with 95% CI during the four different flooding stages of UHE Jirau. Sample-units-based rarefaction (*solid line*) and extrapolation (*dashed line*) plots with 95% CI for *Anopheles* species during the four different flooding stages of UHE Jirau for species richness (**b**), the exponential Shannon index (**c**), and the inverse of the Simpson diversity index (**d**)

Sample coverage for the four stages of flooding (pre-flood, first, second and third stages of flooding) were estimated as being 100, 100, 95.00 and 100%, respectively, indicating that sampling is complete or nearly complete during all stages (Fig. 1a). The smallest number of species (q = 0) was sampled during the first stage of flooding; and no significant difference was found among the other three stages (Fig. 1b). The exponential Shannon index (q = 1) was estimated as 6.89 (CI 6.39; 7.85), 3.86 (CI 3.55; 4.75), 7.55 (CI 6.85; 8.80) and 7.73 (CI 7.30; 8.53) for the pre-flood, first, second and third stages of flooding, respectively (Fig. 1c). This implies that the species richness, when q = 1, is significantly lower during the first stage of flooding. Furthermore, when the inverse of the Simpson diversity index (q = 2) was computed a similar result was found; in this case the values were estimated as 6.57 (CI 5.93; 7.92) during the pre-flood, 3.44 (CI 3.16; 4.53) during the first stage of flooding, 6.89 (CI 6.32; 8.11) during the second stage of flooding and 7.33 (CI: 6.81; 8.60) during the third stage of flooding (Fig. 1d).

During the pre, second and third stages of flooding, all anophelines species were collected; during the first stage of flooding either *An. deaneorum*, *An. konderi*, *An. mattogrossensis,* nor *An. triannulatus* were collected. Thus, a significant difference in species composition was found among the stages of flooding (PERMANOVA: pseudo-F = 3.66, p < 0.05) (Table 2). Thus, when a pairwise comparison (post hoc *test*) was performed, the species composition only differed between the first and second stages of flooding and between the first and third stage of flooding (Table 2).

**Table 2:** PERMANOVA on Bray–Curtis distances matrix for *Anopheles* species composition during the four different flooding stages of UHE Jirau, Porto Velho, Rondonia, Brazil from 2011 to 2015

	DF	Sum of Sq	Mean Sq	F. model	p
Stage	3	0.96	0.32	3.66	0.0008*
Residuals	24	2.09	0.08		
Total	27	3.05			

	t. model	p. adjusted
Pairwise comparison		
First stage of flooding vs pre flood	1.89	0.12
First stage of flooding vs second stage of flooding	2.2	0.01*
First stage of flooding vs third stage of flooding	2.84	0.01*
Pre flood vs second stage of flooding	1.17	1
Pre flood vs third stage of flooding	1.63	0.25
Second stage of flooding vs third stage of flooding	1.14	1

* Indicates statistical difference p < 0.05

**Fig. 2:**
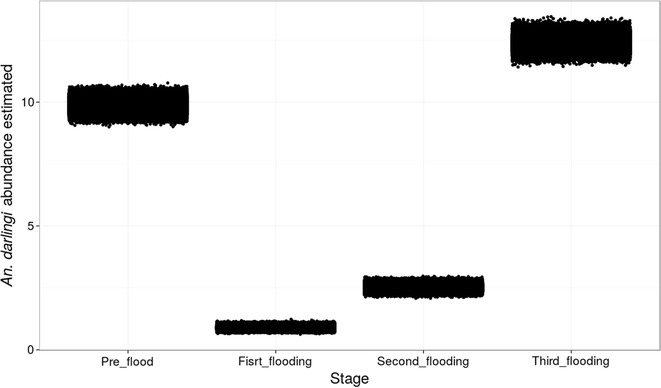
Bayesian estimates of *Anopheles darlingi* abundance in each flooding stage of UHE Jirau, Porto Velho, Rondonia, Brazil from 2011 to 2015. *Points* represent the output of each simulation in the model

The mean abundance of *An. darlingi,* estimated by the model, during the pre-flood was 9.87 (CrI 0.77; 1.01), first stage of flooding 0.88 (CrI 9.49; 10.26), second stage of flooding 2.51 (CrI 2.32, 2.71) and third stage of flooding 12.40 (CrI 11.98; 12.85) (Fig. 2).

## Discussion

Brazil’s anopheline fauna is composed of 54 species, and the importance of a specific species in malaria epidemiology (e.g. primary vector, secondary vector, non-vector) depends on the region in which the species is found. In Brazil, there are currently eight species with a remarkable role in malaria transmission: *Anopheles albitarsis*, *Anopheles aquasalis*, *Anopheles bellator*, *An. braziliensis*, *Anopheles cruzi*, *An. darlingi*, *An. nuneztovari*, and *An. triannulatus* [12–15]. In this study, four of these species were collected, i.e., *An. braziliensis*, *An. darlingi*, *An. nuneztovari* and *An. triannulatus*.

Since northern Brazil is the region with the greatest incidence of malaria, there have been a number of studies of the anopheline fauna of this regions [12, 16, 17]. The anopheline population structure observed in this study is consistent with that reported in the literature. This is to be expected since there is a well-established relationship between the environment and the species that exist therein. Thus, without “any” environmental disturbance no great change in species dynamics is expected in the same place over time [18]. However, as since an environment’s characteristics establish local aspects of the biodiversity like species richness and composition changes in this pattern are expected when a disturbance occurs.

Many species have the ability to modify their own environment; among them, humans are the best example of this behaviour. Several human activities drastically alter the characteristics of an environment, leading to changes in the population structure and dynamic of many others species. Thus, the study of the effect of human activity on biodiversity is common in the literature [5, 19–23]. The effects of habitat alteration on anopheline distribution can be complex, involving changes in species richness, abundance and composition. For example, species with a high degree of resilience may be favoured by the impact and increase in abundance increased. On the other hand, if the species relies highly on the specific characteristics of its habitat it will probably disappear, and, a new species may be introduced in the region. In Amazon, during highway construction, there was a reduction in the abundance of *An. darlingi* due to deforestation which may be what favoured an increase in *An. nuneztovari* abundance. During the construction of the Tucurí dam, *Anopheles argyritasis* appeared in the area; five years later this species was replaced by *An. braziliensis* [4]. Morais et al. [15], studying *Anopheles* fauna near Santo Antônio, saw a reduction in *An. darlingi* abundance, and changes in species richness and composition in the area. However, the authors did not use any ecological measurements in their study which could mislead their data interpretation. De Paula and Gomes [23] reported that there was an increase in anopheline species after the construction of the Porto Primavera dam.

In the present study, a decrease in anopheline richness and differences in species composition during the first stage of flooding were found. After this period, the parameters herein evaluated returned to their original levels (i.e. the same as those observed during the pre-flood stage). Two explanations for this may be hypothesized: (i) most environmental changes occur during the first stage; thus, a decrease in species richness is expected especially from those species that are less abundant in the region [24]; (ii) the construction company adopts strategies to mitigate the impact of the vectors on human health (i.e. insecticides, repellents). Furthermore, an increase in *An. darlingi* abundance during the first stage of flooding and in the third stage of flooding was found. Considering the behaviour of *An. darlingi*, this is to be expected. In this case, since this species has a high degree of anthropophily it can adapt better to an environment affected by human activities compared to other anophelines.

The impact of these changes on malaria epidemiology is not always clear. Changes in the *Anopheles* population (i.e. species richness, composition, abundance and distribution) could lead to changes in host-vector contact, expansion of endemic areas, contact between pathogens and susceptible people, contact between pathogens and potential vectors [25]. One of the first studies linking these topics was conducted by Vittor et al. [26] who found an increase of 278 times in the human biting rate in deforested areas compared to non-impacted areas. One of the components of this measurement is the abundance of the species collected and, in the study cited above, the increase was in *An. darlingi* abundance. A similar result was described by Olson et al. [27], considering malaria cases reported by SIVEP MALÁRIA in deforested areas, and by Moreno et al. [28] in gold mine extraction areas. Monitoring species richness, composition and abundance before, during and after environmental changes could be very useful for public health. For example, the introduction of a new potential vector in a given area could be detected early and control efforts (e.g. insecticides) could be best allocated to avoiding the establishment of the new species.

Unfortunately, there are some limitations in the present studying. First, the sampling points were located near villages and, therefore, anthropophilic species were more likely to be collected than the zoophilic one. Second, neither of the techniques commonly used to search for *Plasmodium* in mosquitoes (i.e. PCR, ELISA or dissection) were used here, nor were any data about cases of malaria, thus, the role of the changes found herein could be linked to changes in the epidemiology of malaria. Third, even if HLC is considered the gold standard method for anophelines, using only one sample technique could lead to bias in estimating the true species that occurs in the region.

## Conclusions

The role of a specific anopheline species in the epidemiology of malaria is not a deterministic process. Furthermore, the ability that some species have to colonize areas where human activities take place may hinder the current vector control programme. Monitoring anopheline population structure, herein considered as species richness, composition and abundance, before, during and after any ecologic alteration in an environment could produce useful data sets that could be used by public health institutions to evaluate the risk of malaria transmission and to help the establishment of effective control and/o r prevention policies for this disease. Furthermore, this approach could improve vector management strategies even in non-impacted areas, since the control efforts adopted by governmental institutions, such as insecticide-treated nets and indoor residual spraying, could drastically change the *Anopheles* community in a given area.

## Additional files

**Additional file 1.** Figures of the studied area. (A) Before the Jirau’s hidroeletric construction (2008); (B) After Jirau’s hidroeletric construction, black line indicates the hidroeletric barrage (2016); (C) Pre flood period, (D) First flooding stage; (E) Second flooding stage; (F) Third flooding stage. Red dots indicates the collection sites. The images were obtained form Google and extracted using Google Earth Pro.

**Additional file 2.** Data used in the analysis.
