# Supplementary figures and images for: Change in Anopheles richness and composition in response to artificial flooding during the creation of the Jirau hydroelectric dam in Porto Velho, Brazil
Source: Malar J. 2017 Feb 22;16:87. doi: 10.1186/s12936-017-1738-7 (PMC5322614; doi:10.1186/s12936-017-1738-7)

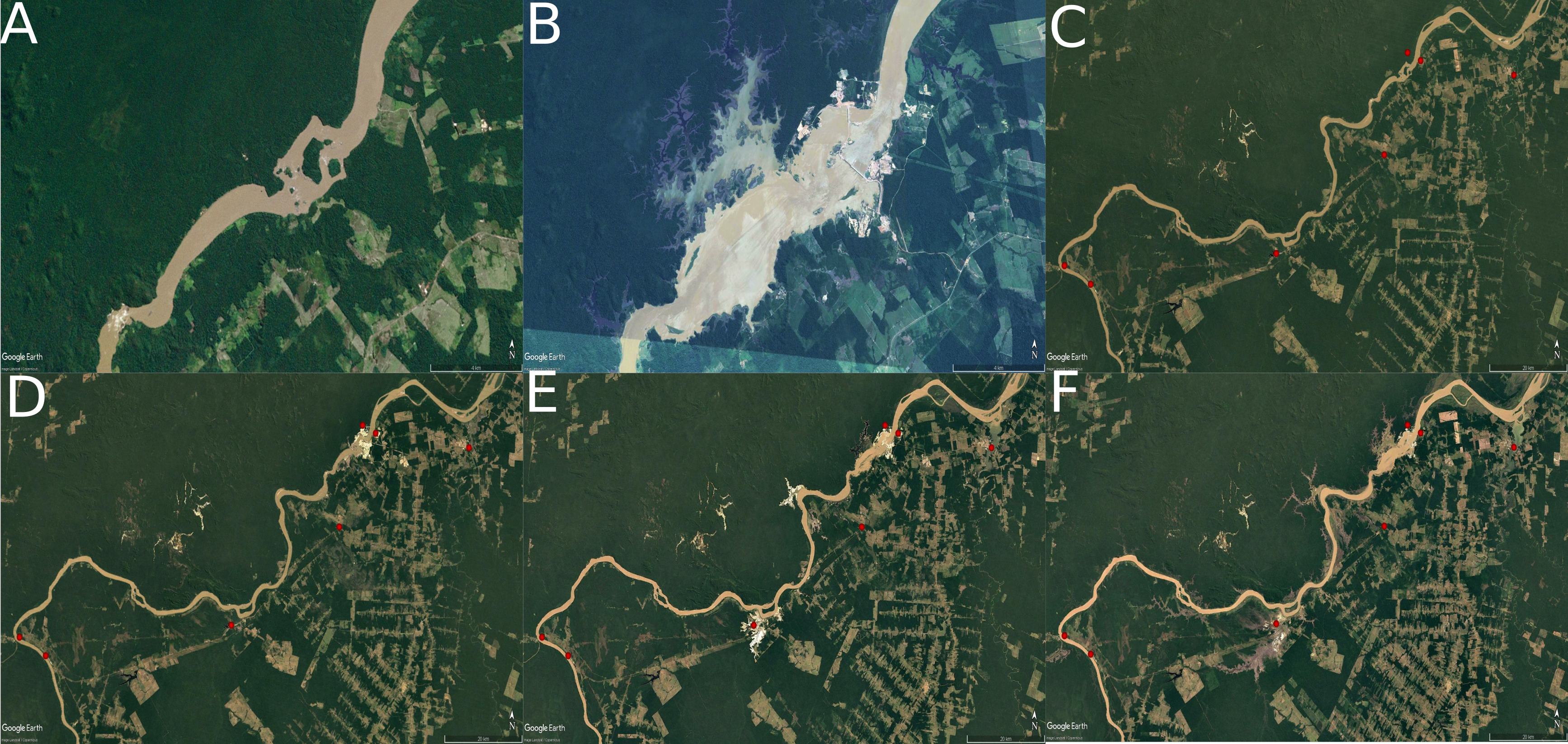

Supplement: Supplementary file 1 — Additional file 1. Figures of the studied area. (A) Before the Jirau’s hidroeletric construction (2008); (B) After Jirau’s hidroeletric construction, black line indicates the hidroeletric barrage (2016); (C) Pre flood period, (D) First flooding stage; (E) Second flooding stage; (F) Third flooding stage. Red dots indicates the collection sites. The images were obtained form Google and extracted using Google Earth Pro. [file 12936_2017_1738_MOESM1_ESM.tiff]
